# Thromboelastography in ruptured abdominal aortic aneurysm management: lessons from vascular, cardiac, and trauma surgery

**DOI:** 10.3389/fcvm.2025.1668165

**Published:** 2025-09-19

**Authors:** Michał Gniedziejko, Joanna Halman, Nina Kimilu, Marcin Folwarski, Mariusz Siemiński

**Affiliations:** ^1^ Emergency Department, University Clinical Centre, Gdańsk, Poland; ^2^ Student Scientific Circle of Neurotraumatology, Medical University of Gdańsk, Gdańsk, Poland; ^3^ Department of Vascular Surgery, University Clinical Centre, Gdańsk, Poland; ^4^ Department of Clinical Nutrition and Dietetics, Medical University of Gdańsk, Gdańsk, Poland; ^5^ Home Enteral and Parenteral Nutrition Unit, Nicolaus Copernicus Hospital, Gdańsk, Poland; ^6^ Department of Emergency Medicine, Medical University of Gdańsk, Gdańsk, Poland

**Keywords:** thromboelastography, TEG, ROTEM, viscoelastic testing, vascular surgery, abdominal aortic aneurysm, rAAA, cardiac surgery

## Abstract

Ruptured abdominal aortic aneurysm (rAAA) is a critical surgical emergency. Thromboelastography (TEG) is a viscoelastic, point-of-care test that provides a comprehensive real-time assessment of coagulation and fibrinolysis. Although TEG has been successfully adopted in trauma and cardiac surgery for individualised transfusion guidance, its role in rAAA has not been sufficiently explored. We conducted a review of studies published between 2009 and 2024 to assess the utility of TEG or rotational thromboelastometry (ROTEM) in rAAA management. Evidence from vascular surgery suggests reduced blood-product transfusions and postoperative bleeding. Additional data from cardiac surgery and emergency/trauma demonstrated improved survival, fewer reoperations and cost efficiency. Although there are few direct studies on the utility of TEG in rAAA and these are mostly descriptive, the results presented in this review suggest its potential role in vascular emergencies. To confirm this, well-designed prospective studies are essential to determine when and how TEG should guide decision-making in this critical setting.

## 1 Introduction

Thromboelastography (TEG) is a non-invasive viscoelastic point-of-care test that evaluates the coagulation process, from clot formation to fibrinolysis. In contrast to standard coagulation tests, which provide limited and often delayed information that is insufficient for targeted transfusion, TEG provides results within minutes, making it particularly valuable in urgent and high-risk surgical situations such as ruptured abdominal aortic aneurysm (rAAA) (1). The role of TEG in vascular emergencies such as rAAA has not yet been sufficiently investigated. Few studies directly evaluate the effects of TEG in vascular surgery, and even fewer in AAA or rAAA. The few available studies are small, heterogeneous and often limited to elective vascular procedures (2–5). In the literature there is lack of standardised, TEG-based transfusion protocols in vascular emergencies. The aim of this review is to summarise the current evidence on the use of TEG in vascular surgery and other surgical specialties, with a focus on its potential application in the treatment of rAAA. To this end, we examine studies from vascular, trauma, and cardiac surgery, where similar bleeding and resuscitation challenges exist, and identify areas where TEG may be beneficial but is not yet routinely used.

## 2 Background

rAAA remains one of the most life-threatening and challenging emergencies in both vascular surgery and emergency medicine. Approximately 60% of patients die before reaching the hospital and total mortality exceeds 80% (6). In recent years, the incidence of rAAA is gradually decreasing in highly developed countries thanks to improved diagnostic methods, better risk stratification, targeted screening programmes, and more effective control of cardiovascular risk factors (7). Nonetheless, these changes do not correlate with a proportional reduction in mortality in patients who usually present to the emergency department in extremis, possibly due to massive haemorrhage, rapidly progressive coagulopathy, acidosis and hypothermia (8, 9). Current treatment strategies include open surgical repair (OSR) and endovascular aneurysm repair (EVAR). In recent years, current guidelines have favoured EVAR over OSR for rAAA when anatomical suitability and local resources allow. Regardless of the technique, rapid control of bleeding and effective resuscitation remain the key determinants of survival (10).

TEG provides comprehensive information on coagulation information within minutes and can differentiate between various causes of coagulopathy (e.g., platelet dysfunction, fibrinogen deficiency or hyperfibrynolisys). This makes it particularly useful in acute situations where rapid, individualised transfusion decisions are required. TEG measures five key parameters: R-time, K-time, α-angle, MA and Ly30 (detailed in Table 1), allowing early detection of coagulopathy and implementation of targeted transfusion protocols. Its usefulness has been confirmed in many areas, including trauma, cardiac surgery and obstetrics, which has been associated with reduced transfusion requirements, fewer reoperations, and lower complication rates (11).

**Table 1:** Key TEG parameters and their clinical interpretation (11).

Parameter	Definition	Clinical interpretation	Normal range
R time	Time until initial fibrin formation (clotting begins); measured from test start to 2 mm amplitude	Reflects coagulation factor activity and initiation of clotting	5–10 min
K time	Time from clot initiation (2 mm) to a fixed clot strength (20 mm amplitude)	Reflects clot kinetics and fibrin cross-linking	1–3 min
α-angle	Angle between baseline and slope of clot formation curve	Indicates speed of fibrin build-up and cross-linking; reflects fibrinogen and platelets	53°–72°
Maximum amplitude (MA)	Maximum strength of the clot	Indicates platelet function and fibrinogen contribution to clot strength	50–70 mm
Ly30	Percentage decrease in amplitude 30 min after MA is reached	Measures degree of fibrinolysis	0%–8%

## 3 Methods

This literature review was based on findings from PubMed, Google Scholar and Web of Science. We included studies conducted from January 2009 to December 2024 and excluded studies outside these dates. Keywords and MeSH terms used included: “thromboelastography”, “TEG”, “ROTEM”, “viscoelastic testing”, “vascular surgery”, “abdominal aortic aneurysm”, “rAAA”, “cardiac surgery”, “trauma”, and “massive transfusion”. We used meta-analysis, observational cohorts, randomized controlled trials, clinical guidelines, expert consensus statements, and case reports. We excluded non-human and *in vitro* models studies, publications not reporting use of TEG or ROTEM and articles not published in English.

## 4 Insights from analogous settings: what cardiac and trauma surgery teach US

### 4.1 Cardiac surgery

Given the limited data on rAAA, it seems reasonable to consider evidence from cardiac surgery, a field where TEG has been widely adopted and validated. In coronary artery bypass grafting (CABG), TEG and platelet mapping (TEG-PM) reduced blood product use (red blood cells - RBC and fresh frozen plasma - FFP), minimised bleeding and led to cost savings up to 45% (12). Similar outcomes were reported by Ak et al., where a kaolin-activated TEG (kTEG) strategy was used in CABG patients. They reported that the kTEG group received 33% less FFP and platelet transfusions and required less tranexamic acid than the clinician-directed group (13). Comparable findings have also emerged from large institutional studies and meta-analyses, including fewer transfusions, fewer reoperations, lower rates of acute kidney injury (AKI), and shorter ICU stays (14–17). Using TEG significantly reduced the transfusion rate of blood products (RBC, FFP, cryoprecipitate) and reduced the number of reoperations due to haemorrhage (14). When comparing ROTEM with standard coagulation laboratory tests (APPT, INR, PLT, fibrinogen, etc.) as a tool to guide transfusion in patients with significant bleeding after cardiac surgery, similar overall transfusion rates, but the ROTEM group had significantly lower 24-hour blood loss (ROTEM: 1538.2 ± 806.4 ml vs. control: 2,056.8 ± 974.5 ml; *p* = 0.032) and lower long-term mortality (ROTEM: 0% vs. control: 15%; *p* = 0.03) in patients with prolonged cardiopulmonary bypass times (15). The meta-analysis conducted by Meco et al. found that TEG or ROTEM protocols significantly reduced RBC and FFP transfusions. It also led to a significant reduction in bleeding 12 and 24 h after surgery, fewer reoperations and a shorter ICU stay (16). In another systematic review and meta-analysis it has been shown that the use of TEG/ROTEM resulted in a 12% reduction in TBC transfusions and a 22% reduction in platelet concentrate transfusions, which was statistically significant. Moreover, a lower incidence rate of AKI was observed in the TEG/ROTEM group (17).

It appears that the findings of the following studies can be applied to the treatment of rAAA, including the need for cardiopulmonary support, fluid volume changes and complex coagulopathy, as cardiac surgery shares several features with vascular surgery. Viscoelastic-guided strategies successfully minimised 24 h blood loss and improved clinical outcomes in patients undergoing prolonged bypass surgery. These conditions closely mimic the physiological derangements observed in rAAA, which are typically associated with persistent hypotension, haemorrhagic shock, and complex coagulopathy.

### 4.2 Trauma surgery

Much more direct are the parallels with trauma surgery. TEG-guided resuscitation in trauma patients has been shown to improve early and 28-day survival, reduce transfusion needs, and allow earlier recognition of coagulopathy. Rapid TEG and ROTEM protocols outperform traditional coagulation lab tests in terms of speed and specificity, which are crucial factors in time-sensitive emergencies such as rAAA (18). Hartmann et al. further advocated for threshold-based, goal-directed transfusion protocols using r-TEG and ROTEM — an approach that could also be used effectively in vascular emergencies. For example, if the clotting time is prolonged, defined as ACT > 140 s (r-TEG) or EXTEM CT > 80 s (ROTEM), then the administration of FFP/PCC is recommended. In case of slow clot formation kinetics characterised by an α-angle <45° (r-TEG) or <63° (EXTEM), cryoprecipitate/fibrinogen should be considered. If the clot strength is also reduced - MA < 48 mm (r-TEG) or MCF < 45 mm (ROTEM)—platelets/fibrinogen should be administered (19).

More insight comes from the study by Reed et al. in which they investigated the utility of viscoelastic tests in 40 emergency department patients with significant bleeding, including gastrointestinal (GI), trauma and rAAA. They documented that the ROTEM A10 test recognized coagulopathy on admission in 25% of trauma patients and 13% of patients with GI haemorrhage on admission, but not in rAAA cases—possibly due to early arrival at hospital or too small sample size. In addition, the ROTEM A10 test provides faster results than standard coagulation tests, enabling earlier intervention (2). Similar results were documented by Cotton et al. They showed that in trauma patients, rapid TEG can predict faster and more precisely the need for transfusion within an hour of admission—early r-TEG and late r-TEG results (within a median time of 5.2 and 14.9 min) were available significantly earlier than conventional coagulation tests (median time of 27 min). These differences emphasise the time-sensitive advantage of TEG in emergency situations (20).

Another study emphasised that viscoelastic tests can predict massive transfusion requirements as early as during triage. It was reported that low MA and α-angle were associated with higher transfusion needs later in the course of treatment (21). The European guideline on the management of major bleeding and coagulopathy following trauma reported that point-of-care strategies such as viscoelastic tests reduce transfusion, transfusion complications (such as TRALI, TACO) and mortality with an average cost reduction per patient of £688 for ROTEM and £721 for TEG compared to conventional coagulation tests (22). Comparable outcomes were described by Whitning et al. The authors observed savings of more than £700 per patient due to fewer ICU days, reduced blood product use, and improved outcomes associated with TEG-guided treatment (23).

### 4.3 Neurosurgery

Beyond trauma, other specialities also offer additional insightful findings. In neurosurgical patients undergoing resection of large primary brain tumors (>4 cm), preoperative, intraoperative and postoperative TEG tests revealed perioperative coagulopathies, typically hypercoagulability, that standard tests failed to detect, guiding tailored transfusion decisions to be made (24). Further evidence of utility of TEG is provided by Neyens et al. They reported the case of an elderly patient taking dabigatran who suffered a subdural haematoma that required urgent surgical treatment. Despite the use of a specific reversal drug, standard coagulation tests indicated severe coagulopathy, while TEG parameters showed adequate clot formation and strength. This illustrates the potential of TEG to support surgical decision-making in anticoagulated patients (25).

### 4.4 Obstetrics

In obstetrics, the utility of viscoelastic testing is well supported by clinical evidence. It has been shown that a TEG-guided algorithm for postpartum haemorrhage (PPH) significantly reduced the need for hysterectomy and lowered the volume of FFP transfused compared to standard management (26). In addition, TEG can detect hypercoagulability more sensitively than standard coagulation tests in pregnant women with pre-eclampsia, particularly on the day of delivery. This suggests its potential utility in both acute and postnatal haemostatic assessment (27). Also coagulation index calculated from TEG parameters can serve as a predictive marker for severe pre-eclampsia when measured in early pregnancy (13–20 weeks) (28). Further support comes from McNamara et al. who applied a ROTEM-based transfusion algorithm for postpartum haemorrhage. This approach significantly reduced both the volume and frequency of blood product use and the incidence of transfusion-related complications (29). These and other findings were summarised in a systematic review,in which TEG/ROTEM were used to detect hypercoagulability during pregnancy to guide transfusion strategies for postpartum haemorrhage, which reduced blood product use compared to standard tests (30).

## 5 Use of TEG in rAAA and vascular surgery: current evidence and opportunities

### 5.1 Use of TEG in vascular surgery

TEG has been successfully used in elective vascular surgery. In systematic review Kim et al. investigated implementation of TEG in cerebrovascular disease, peripheral arterial disease (PAD), deep vein thrombosis (DVT) and arteriovenous malformation (AVM). TEG was successfully implemented to check the stability of carotid plaque and stent outcomes. It assessed bleeding and thrombotic tendencies in patients undergoing surgery for PAD and AVMs. In critically ill and surgical patients, MA levels were used to predict the possibility of DVT and pulmonary embolism (PE), providing physicians with an early indicator of these conditions (31). Further evidence was provided by Cvirn et al. They checked the application of viscoelastic tests in patients undergoing superficial femoral artery (SFA) stenting and documented that a shortened thromboelastometry-derived clotting time (CT) and a higher *α*-angle on ROTEM were associated with a higher risk of in-stent restenosis (ISR). More interestingly, traditional coagulation tests showed no difference between the high and low ISR risk groups. These results are valuable because they show that viscoelastic tests can be used not only to assess intra-operative bleeding and thrombosis management, but also for long-term follow-up of patients with PAD who have undergone stenting procedures (32).

### 5.2 Use of TEG in AAA

TEG is also used in elective AAA repairs. For example Stoneham et al. used TEG in elective AAA repairs with a combination of cell salvage and swab-wash. They showed that in the group of 53 patients, only four required perioperative transfusions. TEG revealed no evidence of coagulopathy or fibrinolysis in these patients at high risk of bleeding, suggesting its value in confirming haemostatic stability and supporting more conservative transfusion strategies. TEG has the potential to be a powerful tool to guide safe transfusion in situations with massive bleeding (3). During thoracoabdominal aortic aneurysm repair, the FIBTEM A10 parameter on the ROTEM has shown utility as both a predictor of severe postoperative bleeding and a guide for fibrinogen supplementation, with a value ≤3 mm serving as a transfusion trigger. These results emphasise the diagnostic power of viscoelastic testing and its potential to tailor interventions to real-time coagulation profiles. Authors emphasise that guidelines based on cardiac surgery are not fully applicable to vascular surgery interventions such as thoracoabdominal aortic aneurysm (TAAA) repair (29). TEG has also been integrated into intraoperative protocols. An example was conducted by Dang et al. in their standardised protocol for ruptured AAAs at Houston Methodist Hospital. TEG was used alongside conventional parameters for haemorrhage monitoring and intraoperative fluid management. Moreover, TEG was proposed as a non-invasive replacement for Swan-Ganz catheters in mechanically ventilated patients to guide fluid and blood-product transfusions (5).

Overall, these findings show that TEG play an important role in individualised transfusion and coagulation management in vascular surgery and thanks to that, cost-effectiveness through fewer transfusions, reoperations and better outcomes. While these results are promising, they are mostly limited to elective procedures. rAAA presents a fundamentally different scenario characterised by haemodynamic instability, active bleeding and time-critical decisions.

Unfortunately, there are few studies specifically investigating TEG in rAAA. In one emergency department study, ROTEM examination of five rAAA patients did not reveal coagulopathy on admission, though this may reflect early presentation rather than absence of dysfunction. Nevertheless, the speed of ROTEM (A10 available in a few minutes compared to 57 min for traditional coagulation tests) supports its potential for early triage and management (2). Summary of included studies evaluating TEG/ROTEM in Vascular, Cardiac andTrauma Surgery is presented in Table 2.

**Table 2:** Key characteristics of included studies evaluating TEG/ROTEM in vascular, cardiac, and trauma surgery.

Specialty	Study type	*n*	Setting	TEG/ROTEM use	Key outcome(s)	Study (Author, Year)
Cardiac (CABG)	RCT	249	Pre- and post-operative	TEG	Reduced transfusion requirements, reduced bleeding, and cost savings	Agarwal et al., 2020 (12)
Cardiac (CABG)	RCT	224	Intraoperative	TEG	Reduced transfusion requirements	Ak et al., 2009 (13)
Cardiac (CABG)	Cohort	681	Perioperative	TEG	Reduced transfusion requirements, reduced bleeding	Fleming et al., 2017 (14)
Cardiac	RCT	80	Postoperative	ROTEM	Reduced transfusion requirements, reduced bleeding, improved outcomes	Haensig et al., 2019 (15)
Cardiac	meta-analysis	1,035	Various	TEG/ROTEM	Reduced transfusion requirements, reduced bleeding, shorter ICU stay	Meco et al., 2020 (16)
Cardiac	Systematic review and meta-analysis	8,723	Various	TEG/ROTEM	Reduced transfusion requirements, reduced bleeding, lower incidence of AKI	Serraino and Murphy, 2017 (17)
Trauma	RCT	111	ED	TEG (rapid TEG)	Improved 28-day survival, reduced transfusion requirements, shorter ICU stay	Gonzalez et al., 2016 (18)
Trauma	Narrative review/Expert summary	–	Various	TEG/ROTEM	Tresholds for guided transfusion protocols	Hartmann et al., 2020 (19)
Emergency (rAAA, trauma, GI bleeding)	Observational	40	ED	ROTEM (A10 within 10 min)	Faster triage, no coagulopathy in rAAA	Reed et al., 2013 (2)
Emergency	Observational	272	ED	TEG (rapid TEG)	Predicted early transfusions needs	Cotton et al., 2011 (20)
Emergency	Review/Expert Consensus Statement	–	ED	TEG/ROTEM	Predicted massive transfusions,	Brill et al., 2021 (21)
Emergency	Guidelines	–	Various	TEG/ROTEM	Reduced transfusion requirements, complications, mortality	Spahn et al., 2019 (22)
Multidisciplinary	Systematic review, cost-effectivness analysis	7,092	Various	TEG/ROTEM	More cost-effectiveness (because of reduced transfusion requirements, shorter ICU stay, better outcome)	Whiting et al. 2015 (23)
Neurosurgery	Observational	40	Pre-, intro- and post-operative	TEG	Identified hypercoagulability	Khatri et al., 2021 (24)
Neurosurgery/Emergency	Case report	1	ICU	TEG	TEG confirmed adequate clot formation and enabled safe subdural drain insertion despite CCTs were still abnormal	Neyens et al., 2014 (25)
Obstetrics	Open controlled trial	119	Intraoperative	TEG	Significantly reduced hysterectomy rate, reduced blood loss and transfusions requirements	Barinov et al., 2015 (26)
Obstetrics	Cohort	59	Delivery, 6 weeks and 6 months postpartum	TEG	TEG revealed hypercoagulability in preeclampsia missed by conventional labs; normalized by 6 weeks postpartum	Murray et al., 2018 (27)
Obstetrics	Observational	184	13–20 and 35–40 weeks of pregnancy	TEG	Coagulation index <0 during early pregnancy strongly predicted severe preeclampsia	Wang et al., 2019 (28)
Obstetrics	Cohort	255	Maternity ward	ROTEM	Reduced blood product use and ICU admission; no TACO; individualized therapy superior to shock packs	McNamara et al., 2019 (29)
Obstetrics	Systematic review, expert recommendation	32,817	Various	TEG/ROTEM	Assessment of hypercoagulability in obstetrics patients, guided-transfusion in postpartum hemorrhage	Amgalan et al. 2020 (30)
Vascular (Elective AAA)	Cohort	53	Intraoperative	TEG with cell salvage	Only 4 patients required transfusion	Stoneham et al., 2023 (3)
Vascular (TAAA)	Cohort	166	Intraoperative	ROTEM (FIBTEM A10)	Trigger for fibrinogen supplementation	Monaco et al., 2021 (4)
Vascular (AAA)	Guideline/Case series	–	Intraoperative	implementation of TEG to guide transfusion in rAAA	Reduced bleeding, individualized resuscitation	Dang et al., 2023 (5)
Vascular (cerebrovascular diseases, PAD, AVM)	Systematic Review	∼7328	Various	TEG in vascular surgery	Successful implementation of TEG in patients with cerebrovascular diseases, PAD, AVM	Kim et al., 2022 (31)
Vascular (PAD)	Observational	34	post-operative	ROTEM	Predicted in-stent restenosis	Cvirn et al., 2012 (32)

AAA, abdominal aortic aneurysm; TAAA, thoracoabdominal aortic aneurysm; PAD, peripheral artery disease; CABG, coronary artery bypass grafting; RCT, randomized controlled trial; ED, emergency department; AVM, arteriovenous malformation; ICU, Intensive Care Unit; GI, gastrointestinal. CCT refers to conventional coagulation tests such as PT, prothrombin time; aPTT, activated partial thromboplastin time; INR, international normalized ratio and fibrinogen level.

## 6 Limitations

The main limitation of this review is the lack of reliable evidence, especially in the field of emergency medicine. Most of the available data are from observational studies or extrapolated from trauma, cardiac, and elective vascular surgery. In addition, the heterogeneity of TEG/ROTEM protocols, transfusion thresholds, and patient populations across studies limits comparability. Finally, the lack of standardised TEG-guided algorithms specifically tailored to vascular emergencies highlights the need for prospective studies to evaluate clinical effectiveness in this area. To address this gap, there is a clear need for well-designed prospective studies and randomised controlled trials specifically investigating how TEG can guide transfusion strategies in rAAA patients. Key outcomes to examined should include short- and long-term mortality (e.g., 24 h and 30 days), volume of blood products used, time to hemostasis, transfusion-related complications and overall cost-effectiveness. It would also be valuable to explore whether early TEG performed early, ideally on arrival in the emergency department, could help identify patients at higher risk of bleeding and adjust their resuscitation accordingly. Given how quickly the condition of these patients can deteriorate and how common coagulopathy is in this situation, there is a strong rationale for including TEG in massive transfusion protocols. These protocols could be adapted from existing trauma and cardiac models, but should be customized to reflect the unique challenges of rAAA.

## 7 Conclusion

rAAA is characterised by severe haemodynamic instability and massive bleeding. This review shows TEG offers significant advantages in the management of coagulopathy in complex bleeding events. Evidence from trauma, cardiac, elective vascular surgery and other surgical specialities suggests that viscoelastic tests such as TEG and ROTEM have proven to be useful to guide patient management in different surgical specialities. Compared to standard tests, they provide faster results and offer much more comprehensive information on coagulopathy, especially in haemodynamically unstable patients. This has led to improved clinical outcomes, including reduced blood product use, shorter ICU stays, lower mortality, fewer complications and lower costs per patient. Although the current evidence for the use of TEG in rAAA is limited, evidence from related specialities supports its integration into emergency protocols. However, further research is needed to validate the benefits of TEG in the context of rAAA and to define its optimal role. Ultimately, the development of standardised, TEG-guided resuscitation pathways in vascular surgery centres could support more personalised care, reduce transfusion requirements and improve survival in these high-risk patients.
